# Gallbladder Tuberculosis Presenting as Obstructive Jaundice

**DOI:** 10.14309/crj.0000000000000414

**Published:** 2020-06-25

**Authors:** Bharti Varshney, Poonam Elhence, Subhash Soni, Vaibhav Kumar Varshney, Taruna Yadav, Souvik Saha

**Affiliations:** 1 Department of Pathology, All India Institute of Medical Sciences, Jodhpur, Rajasthan, India; 2 Department of Surgical Gastroenterology, All India Institute of Medical Sciences, Jodhpur, Rajasthan, India; 3 Department of Diagnostic and Interventional Radiology, All India Institute of Medical Sciences, Jodhpur, Rajasthan, India

## Abstract

Isolated gallbladder tuberculosis is a rare entity, even in endemic zones. Preoperative diagnosis is usually not possible, with most of the cases being diagnosed either as cholecystitis or malignancy. Histopathological examination of the resected specimen clinches the diagnosis. We present a middle-aged man with obstructive jaundice who was diagnosed to have gallbladder malignancy clinically and radiologically and on microscopy, and was diagnosed as gallbladder tuberculosis with the involvement of the common bile duct.

## INTRODUCTION

Gallbladder involvement with tubercular bacilli in isolation is extremely rare.^1^ Preoperatively, most patients are misdiagnosed as chronic cholecystitis or gallbladder malignancy.^2–4^ The patient presented with jaundice and clinicoradiological features favoring malignancy, after which radical cholecystectomy was performed. On microscopy, diagnosis of gallbladder tuberculosis (GBTB) involving the common bile duct (CBD) was given. Postoperatively, the patient underwent relevant investigations and repeat meticulous clinical examination, and tuberculosis (TB) of other sites was excluded. Isolated GBTB extending to the CBD and presenting as obstructive jaundice has not been reported so far.

## CASE REPORT

A 55-year-old man presented with persistent pain in the right upper abdomen for 6 months, relieved with medication, which recurred. After 4 months, he noticed progressive jaundice, clay-colored stools, itching, significant weight loss, and anorexia. There was no history of awareness of lump, abdominal distension, or chronic cough. On general examination, he was icteric and had no lymphadenopathy. Abdominal examination revealed palpable gallbladder, which was smooth and firm.

**Figure 1.**
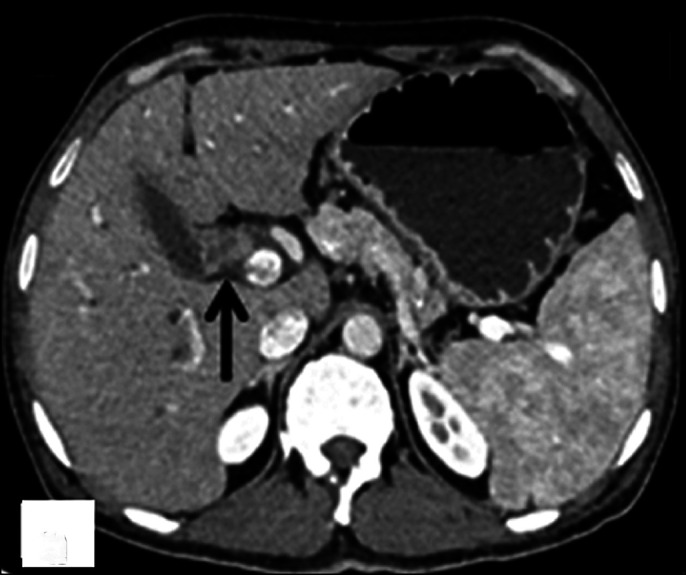
Abdominal contrast-enhanced computed tomography showing thickening at the gallbladder neck with extension along cystic duct and proximal common bile duct (arrow).

**Figure 2.**
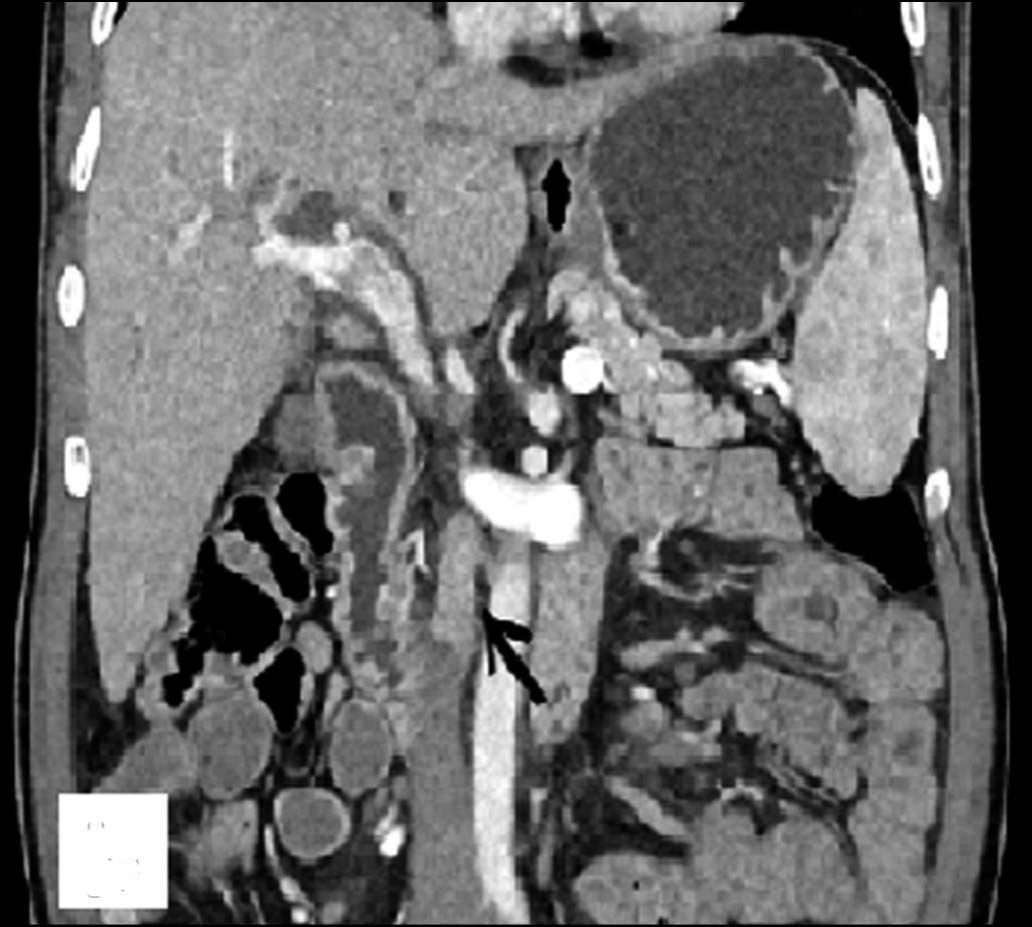
Homogeneously enhancing interaortocaval lymph node (arrow).

**Figure 3.**
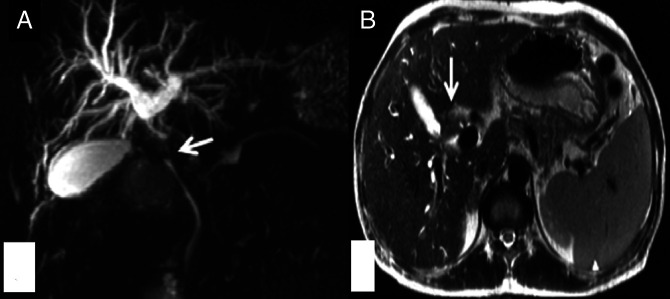
Magnetic resonance cholangiopancreatography image showing (A) complete block at the level of the common bile duct (white arrow) with moderate bilobar intrahepatic biliary radical dilatation and (B) axial T2W1 depicting T2 hypointense wall thickening of the gallbladder neck (white arrow).

The patient's serum total bilirubin was 6.93 mg/dL with direct component of 4.26 mg/dL, serum aspartate aminotransferase was 175 U/L, serum alanine aminotransferase was 153 U/L, and serum alkaline phosphate was 1100 U/L. Abdominal ultrasound scan showed echogenic soft-tissue thickening along the gallbladder neck and proximal CBD with dilatation of intrahepatic biliary radicals. Contrast-enhanced computed tomography showed enhancing thickening at the gallbladder neck (up to 5.5 mm) with periductal spread along the cystic duct and proximal CBD causing its obstruction and proximal intrahepatic biliary radical dilatation (Figure 1). Multiple homogeneously enhancing discrete lymph nodes were present in periportal, precaval, and interaortocaval regions without any compression of the biliary system (Figure 2). Magnetic resonance cholangiopancreatography images showed complete block at the level of the CBD with moderate bilobar intrahepatic biliary radical dilatation and axial T2W1 image depicting T2 hypointense wall thickening of the gallbladder neck (Figure 3). This thickening showed restriction on diffusion-weighted images. A diagnosis of gallbladder malignancy with likely periductal spread to the CBD was made. Preoperative image-guided biopsy was not performed because of the high risk of tumor seeding in malignancy.

Diagnostic laparoscopy showed distended gallbladder with thickening involving the cystic duct and CBD, and reaching just below the confluence. Frozen section of interaortocaval lymph nodes revealed granulomatous inflammation and no evidence of malignancy. Hence, radical cholecystectomy with excision of CBD and Roux-en-Y hepaticojejunostomy was performed.

**Figure 4.**
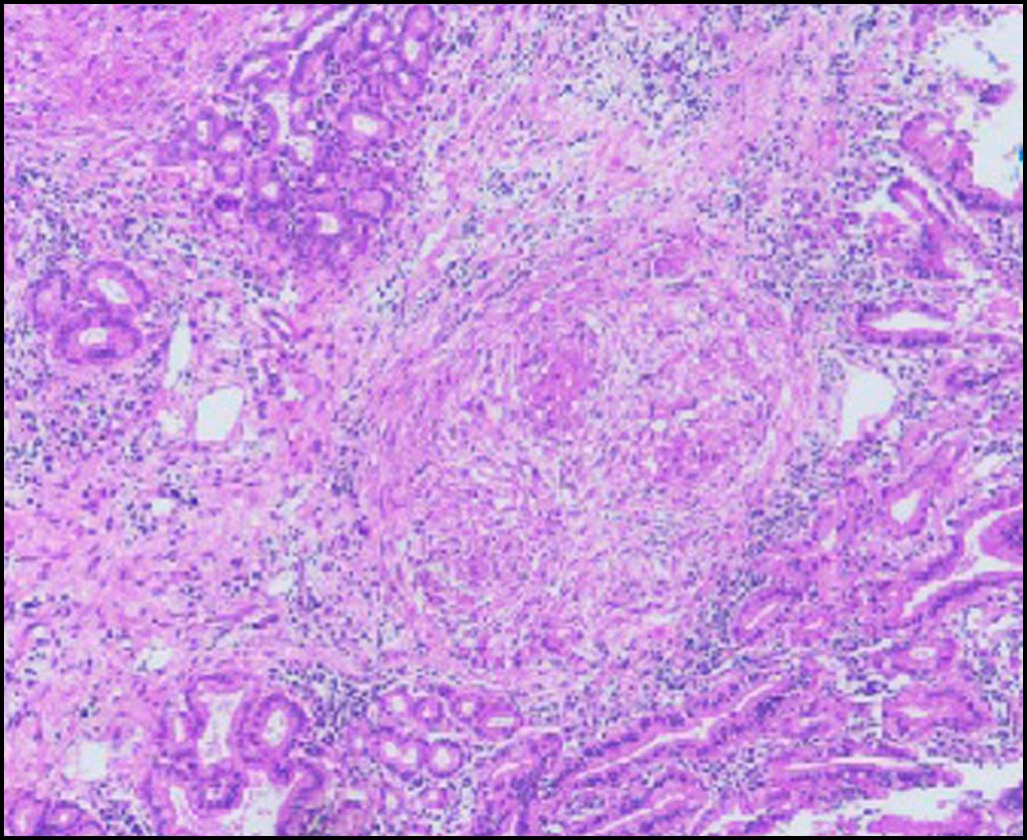
Microphotograph of the gallbladder showing gallbladder mucosa and epithelioid cell granulomas (hematoxylin and eosin stain, 4× magnification).

**Figure 5.**
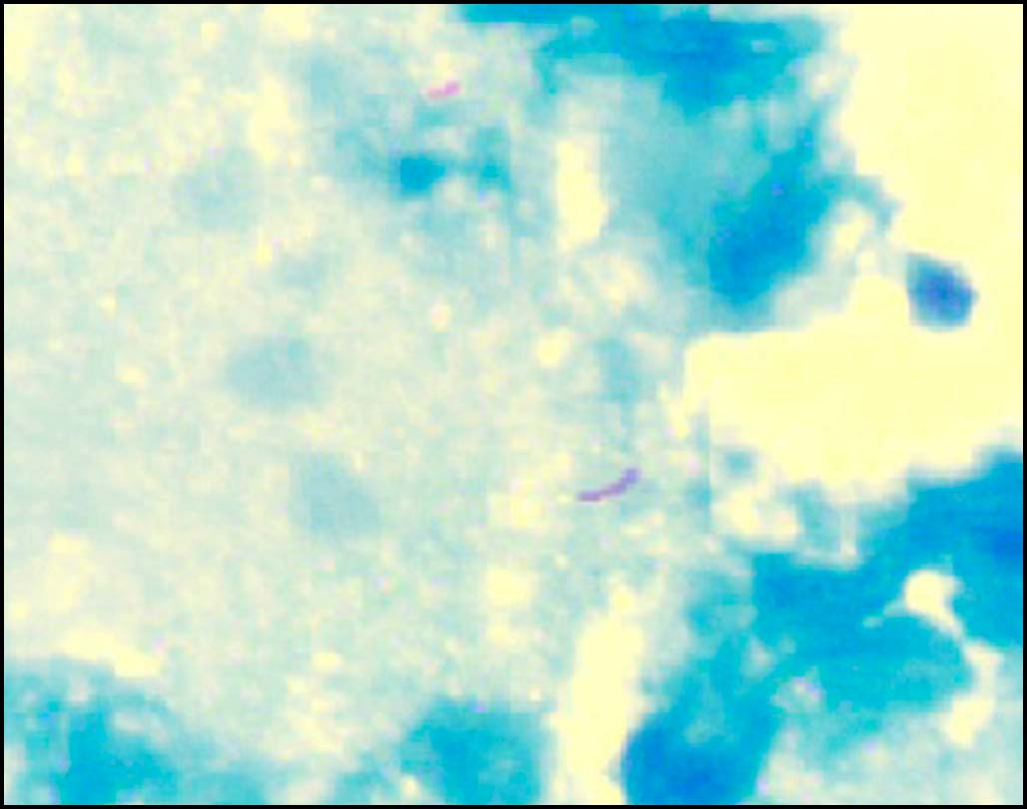
Microphotograph of the gallbladder showing the presence of acid-fast bacilli (Ziehl-Neelsen stain, 100× magnification).

Microscopy of the gallbladder revealed extensive caseous necrosis and numerous epithelioid cell granulomas with Langhans giant cells (Figure 4). Lymph nodes and CBD resection margin showed similar microscopic features. Ziehl-Neelsen (ZN) stain showed acid-fast bacilli confirming TB (Figure 5). Sections from the liver bed showed no granulomas or necrosis. A diagnosis of necrotizing tubercular cholecystitis involving the proximal CBD was rendered.

In the postoperative period, the patient was allowed oral intake on the second postoperative day (POD). Abdominal drain was removed on the third POD, and he was discharged on the fifth POD. At the time of discharge, his serum total bilirubin was 1.56 mg/dL with direct of 0.72 mg/dL. Retrospectively, TB infection from the abdomen and chest were excluded. Immune-compromised status was ruled out. Antitubercular therapy with isoniazid (I), rifampicin (R), ethambutol (E), and pyrazinamide (P) for 6 months (intensive phase: 2 months of all 4 drugs and continuation phase: 4 months of E & P) was started. After the 9-month follow-up, he is doing well.

## DISCUSSION

Hepatobiliary TB accounts for nearly 1% of abdominal TB.^1,5^ GBTB is uncommon because high alkalinity of bile and bile acids prohibit the growth of tubercle bacillus.^6^ However, cystic duct obstruction causing low bile acid concentration and damage to the gallbladder mucosa predisposes to GBTB. TB can involve the gallbladder by hematogenous or lymphatic route from adjacent positive foci, by serosal spread from a peritoneal lesion or canalicular dissemination.^6^ In the present case, the blockage of the cystic duct most likely predisposed to tubercular infection. Furthermore, cholelithiasis, which acts as a nidus for the development of TB, has been reported in nearly two-thirds of patients with GBTB,^6^ but it was not noted in the present case.

GBTB has myriad presentations as a component of miliary or disseminated abdominal TB in immunocompromised states or as isolated involvement,^7^ as seen in the present case. The symptomatology may mimic acute cholecystitis to frank malignancy.^2–4^ Hence, GBTB should be considered as a differential diagnosis for gallbladder mass, especially in individuals from an endemic area or in an immunocompromised patient.

Preoperatively, GBTB poses a diagnostic dilemma despite advanced imaging modalities, leading to missed diagnosis. Contrast-enhanced computed tomography imaging of GBTB may show thickened gallbladder wall, nodular lesion with homogeneous enhancement, calcific flecks, and necrosis.^5^ The existing literature for biliary TB on magnetic resonance imaging (MRI) is scarce and mostly described as biliary strictures.

On a retrospective review of MRI findings of this case, we feel that T2 hypointense nature, diffusion restriction, and peripheral enhancement of soft-tissue thickening could have been a clue for a differential of biliary TB. Because biliary malignancies with sclerosing component can show similar features, diagnosis of gallbladder malignancy with periductal spread was made. We suggest that all these findings well described for hepatic tuberculomas^8^ can be extrapolated to biliary tubercular lesions as demonstrated in our case.

Various peculiarities in the present case distinguish it from previously reported cases. First and foremost, the rare presentation of GBTB extending from the cystic duct into CBD leading to obstructive jaundice has not been reported so far. Secondly, the usual causes of obstructive jaundice in context of abdominal TB or GBTB are due to CBD compression by pericholedochal lymph nodes.^4^ In the present case, the lymph nodes showed granulomas but not compressing the CBD. Thirdly, GBTB is preferentially noted in young women.^1,5^ In the present case, the patient is an older man. Finally, most of the literature mentions classical microscopic findings suggesting the diagnosis of TB. However, documentation of *Mycobacterium tuberculosis* on ZN stain has only been reported once,^2^ but image for the same was not shown. The current case demonstrates acid-fast bacilli on ZN stain.

This case highlights that biliary TB should be included in the preoperative imaging differentials on MRI despite its rarity. This report reiterates the value of meticulous histopathological examination of all gallbladder specimens.

## DISCLOSURES

Author contributions: B. Varshney, P. Elhence, S. Soni, T. Yadav, and S. Saha wrote the manuscript. B. Varshney, P. Elhence, and VK Varshney revised the manuscript for intellectual content and approved the final manuscript. P. Elhence is the article guarantor.

Financial disclosure: None to report.

Informed consent was obtained for this case report.
